# Serum CCL21 as a Potential Biomarker for Cognitive Impairment in Spinal Cord Injury

**DOI:** 10.1155/2020/6692802

**Published:** 2020-12-09

**Authors:** Yuanzhen Chen, Liangke Liang, Shengnan Cao, Guangjian Hou, Qian Zhang, Hong Ma, Bin Shi

**Affiliations:** ^1^Neck-Shoulder and Lumbocrural Pain Hospital, Shandong First Medical University & Shandong Academy of Medical Sciences, Jinan, 250014 Shandong Province, China; ^2^Foshan Traditional Chinese Medicine Hospital, Foshan, 528000 Guangdong Province, China; ^3^School of Acupuncture-Tuina, Shandong University of Traditional Chinese Medicine, Jinan, 250355 Shandong Province, China; ^4^Taian City Central Hospital, Taian, 271000 Shandong Province, China

## Abstract

**Objective:**

Cognitive impairment is considered to be an important complication of spinal cord injury (SCI), but its underlying mechanism remains unclear. The purpose of this study is to explore whether serum CCL21 can be used as a potential biomarker of cognitive impairment in SCI.

**Methods:**

In Neck-Shoulder and Lumbocrural Pain Hospital, Shandong First Medical University & Shandong Academy of Medical Sciences, hospitalized or treated acute SCI patients were included in the study as the SCI group (SCI). At the same time, a normal control group (NC) matching the age and sex of the SCI group was recruited in the outpatient clinic. Once the two groups were enrolled, their demographics and clinical characteristics were collected immediately. Enzyme-linked immunosorbent assay (ELISA) was used to detect serum CCL21 levels within 24 hours of admission. Three months later, the Montreal Cognitive Assessment (MoCA) was used to test the cognitive function of the population.

**Results:**

A total of 84 SCI patients and 49 NC populations were eligible for inclusion in the study. There was no significant statistical difference in the demographics and clinical characteristics (age, gender, BMI, TG, LDL-C, FBG, SBP, and DBP) between the two groups (*p* > 0.05). Compared with the NC group, the SCI group had a higher serum CCL21 level (*p* < 0.001) and a lower MoCA score (*p* < 0.001). Serum CCL21 level in SCI was negatively correlated with MoCA score (*p* = 0.023). Multivariable analyses showed that serum CCL21 level is an independent prognostic factor of cognitive impairment in SCI.

**Conclusions:**

MoCA score has a linear relationship with serum CCL21 quartile, and SCI cognitive function has a negative correlation with serum CCL21. Serum CCL21 is an independent risk factor for cognitive impairment after SCI.

## 1. Introduction

Spinal cord injury (SCI) can be defined as the injury caused by complete or incomplete damage to the motor function, sensory function, autonomic nerve, and reflex after the spinal cord is injured [1]. SCI is a common and highly destructive neurological disease, which has a profound impact on society from the perspectives of physiology, psychology, economy, family, and culture [2, 3]. The incidence of SCI is on the rise globally, with an estimated incidence of 10.4-83 per million per year. The incidence of SCI is 20.7-83.0 in North America, 8.0-130.6 in Europe, and 23.7-60.6 in China [4]. As there is currently no effective treatment for SCI, patients with SCI have to bear a considerable economic burden in terms of treatment and rehabilitation [5]. Among the many complications of SCI, cognitive impairment has received more and more attention in recent years. According to reports, 40-60% of patients with SCI may have varying degrees of cognitive impairment [6]. However, the mechanism of cognitive impairment after SCI remains unclear.

Chemokines are chemotactic cytokines that coordinate the localization of cells including immune cells [7]. Chemokines play a key role in various processes, including the development and homeostasis of immune cells, the initiation of innate and adaptive immune responses, and the recruitment of immune cells under pathological conditions [8]. CCL21 is an effective microglia-activating chemokine, which is synthesized by damaged neurons, transported by axons, and then released to activate microglia [9]. CCL21 is only expressed in injured neurons in the central nervous system and can quickly cause neuroinflammation in the injured local and remote sites [10]. CCL21 in vivo mainly plays a pathophysiological role through two receptors: CCR7 and CXCR3 [11]. However, the mechanism of CCL21 involved in nerve damage is still unclear, and it is a hotspot for future research.

The possible mechanism for the high risk of dementia in SCI patients is posttraumatic neuroinflammation and related neurodegeneration [12]. Chemotactic cytokines are important factors that initiate and participate in the inflammatory process [13]. The role of CCL21 in SCI has received considerable attention in recent years. The purpose of this study is to explore whether serum CCL21 can be used as a potential biomarker of cognitive impairment in SCI.

## 2. Methods

### 2.1. Study Population

Acute SCI patients who were treated at the Neck-Shoulder and Lumbocrural Pain Hospital, Shandong First Medical University & Shandong Academy of Medical Sciences, from March 2017 to February 2020 were included in this study. The diagnosis of SCI is based on the diagnostic criteria of the guidelines [14, 15]. Enrollment criteria are as follows: (1) SCI patients within 24 hours of onset; (2) 18-80 years old; and (3) able to complete relevant examinations. Exclusion criteria are as follows: (1) head injury; (2) unconsciousness or history of mental illness; (3) inflammatory disease or autoimmune disease; (4) suffering from cognitive dysfunction such as Alzheimer's disease (AD) and vascular dementia (VaD); (5) tumor; and (6) unable to complete related examinations. The study was approved by the local ethics committee. The research subjects were informed of the study, and informed consent was signed by them or their guardians.

### 2.2. Demographics and Clinical Characteristics

Once enrolled in the group, the demographics and clinical characteristics of the study population are registered. The demographics and clinical characteristics include age, gender, body mass index (BMI), triglycerides (TG), low-density lipoprotein cholesterol (LDL-C), fasting plasma glucose (FBG), systolic blood pressure (SBP), and diastolic blood pressure (DBP). Questionnaires are used to collect demographic data. The clinical characteristics are obtained by applying standard laboratory testing methods.

### 2.3. Serum CCL21 Levels

Collect 5 ml of fasting venous blood from all study populations within 24 hours of admission. The sample should be collected in a serum separation tube. After clot formation, centrifuge at 2000 × g for 10 minutes to collect serum. The sample is diluted into sample diluent NS and measured. Store the undiluted serum in aliquots at -20°C or below for later use. Enzyme-linked immunosorbent assay (ELISA) was used to detect the level of serum CCL2. CCL2 antibody is a purchased commercial reagent (Abcam, MA, USA). The steps of ELISA refer to previous reports and reagent instructions [16]. Record the OD at 450 nm under a spectrophotometer.

### 2.4. Cognitive Function Evaluation

The evaluation of cognitive function uses the Montreal Cognitive Assessment (MoCA). MoCA is a widely used screening tool to detect cognitive impairment, which was created by Ziad Nasreddine in Montreal in 1996. MoCA has good sensitivity and specificity and is especially suitable for the detection of mild cognitive impairment. It has been widely used in many countries. The MoCA test is a test with a total score of 30 points and is completed in approximately 10 minutes. A score of 26 or more is considered normal [17]. The evaluation of MoCA is completed by professionally trained physicians, who are blind to the grouping and baseline data of the study population.

### 2.5. Statistical Analysis

The quantitative data of the normal distribution is expressed by the mean ± standard deviation (SD), and the qualitative data is expressed by the rate. The comparison of quantitative data uses *t*-test, and the comparison of qualitative data uses nonparametric test. According to the quartile of serum CCL21, subjects were divided into four groups with the same sample size. Multivariable analyses were used to explore independent predictors of cognitive impairment after SCI. The SPSS version 20.0 (SPSS, Chicago, IL, USA) was used for statistical analysis, and *p* < 0.05 was considered statistically significant.

## 3. Results

### 3.1. Demographics and Clinical Characteristics

A total of 133 subjects were recruited for this study, including 49 NC and 84 SCI patients. The demographics and clinical characteristics are shown in Table 1. In terms of age, gender, BMI, TG, LDL-C, FBG, SBP, and DBP, there was no significant difference between the NC group and the SCI group (*p* > 0.05). However, the CCL21 levels of the NC group and SCI group were 41.3 ± 5.2 pg/ml and 119.7 ± 13.5 pg/ml, respectively. The CCL21 level of the SCI group was significantly higher than that of the NC group, and there was a significant statistical difference between the two groups (*p* < 0.05). In addition, the MoCA scores of the NC group and SCI group were 28.2 ± 1.2 points and 24.6 ± 1.9 points, respectively. The MoCA score of the SCI group was significantly lower than that of the NC group, and there was a significant statistical difference between the two groups (*p* < 0.05).

### 3.2. Relationship between Serum CCL21 and Cognitive Function in SCI

According to the serum CCL21 quartile, the MoCA scores of SCI patients are shown in Table 2. The results showed that the MoCA score of SCI patients had a linear relationship with the quartile of serum CCL21; that is, the MoCA score of SCI patients decreased with the increase of serum CCL21 level (*p* = 0.023).

### 3.3. Independent Risk Factors for Cognitive Impairment with Multivariable Analyses in SCI

The results of multivariable analyses are shown in Table 3. Multivariable analyses showed that serum CCL21 is an independent predictor of cognitive impairment after SCI (*β* = 0328, *p* = 0.027). After adjusting for confounding factors such as age, gender, BMI, TG, LDL-C, FBG, SBP, and DBP, serum CCL21 still has a predictive effect on cognitive impairment after SCI.

## 4. Discussion

The main finding of the current study is that compared with the NC group, the serum CCL21 level in the SCI group was significantly increased, and the serum CCL21 level was negatively correlated with the MoCA score. Further research found that serum CCL21 can be used as a potential biomarker for the diagnosis of cognitive impairment after SCI. Our study confirmed for the first time the correlation between serum CCL21 and the prognosis of cognitive function in patients with SCI.

More and more evidences show that cognitive impairment is a complication of SCI. Zhang et al. observed mitochondrial swelling and vacuolation of the endoplasmic reticulum in the hippocampus in a pig thoracic spinal cord injury model, accompanied by a decline in learning and memory [18]. This is the first animal experiment to confirm the correlation between spinal cord injury and deterioration of brain cognitive function. In addition, in the rodent SCI model, we can not only observe long-term deficits in motor function but also gradually lose cognitive ability after 8 weeks of modeling [19, 20]. Not only in animal models but also in human cognitive impairment after SCI has been reported in recent years. A US study of SCI patients in residential communities showed that after SCI, the risk of cognitive impairment is increased, mainly impairing patients' processing speed and executive function for specific tasks [21].

Although cognitive impairment after SCI has been widely reported, its underlying pathophysiological mechanism remains unclear [22]. SCI produces cell debris and releases intracellular proteins, which can induce a strong inflammatory response. In an inflammatory state, resident inflammatory cells, including astrocytes and microglia, are rapidly activated to release a variety of inflammatory mediators such as regulatory factors, cytokines, chemokines, and growth factors [23]. The release of chemokines and cytokines can recruit surrounding neutrophils and macrophages into the injured spinal cord. The first wave of infiltrating immune cells is neutrophils. Neutrophils have a bactericidal effect and are considered to be the first line of defense against invaders [24]. They are also considered to be the main contributor to SCI pathological damage. A Canadian study showed that the expression of IL-1*β*, an inflammatory factor in the central nervous system, can increase 15 minutes to 3 hours after SCI, and it can last for 24 hours. This inflammatory response was weakened in the IL-1*β* gene knockout mouse model of SCI, confirming that inflammation is involved in the acute injury of SCI [25]. A recent study showed that the expression of CD200 was downregulated after SCI, thereby attenuating the signal transduction of the CD200-CD200R1 axis and increasing neuroinflammation [26]. The above studies suggest that intervention of inflammation may be an important means to improve the prognosis of SCI.

CCL21 is an effective microglia-activating chemokine, and its role in SCI has recently been reported. Research by Honjoh and his colleagues showed that compared with wild-type mice, the number of classically activated (M1 phenotype) microglia in mice with low expression of CCL21 gene mutations in the SCI model was significantly reduced [27]. This study suggests that CCL21 may be an important target for intervention in neuroinflammation after SCI. Research by Zhao et al. showed that after SCI, it can induce the upregulation of the neuroimmunomodulator CCL21 in the thalamus, thereby further enhancing the microglia-related inflammatory response associated with pain phenomena [28]. However, the specific mechanism of CCL21 involved in nerve damage after SCI is still unclear.

CCL21 has been reported in animal studies on cognitive impairment after SCI [20]. In different degrees of SCI mouse models, the upregulation of CCL21 and the activation of microglia can be seen, as well as neuron loss, hippocampal neurogenesis, and cognitive decline. The accumulation of CCL21 in the brain may be a potential target of cognitive impairment after SCI. However, our study confirmed the correlation between CCL21 and cognitive function in human SCI for the first time. This is the strength of our research. However, our research has limitations. First, this is a single-center study with a small sample; second, we did not monitor the dynamic changes of serum CCL21; and third, we did not collect information on the subjects' medications, which may interfere with our research results.

## 5. Conclusions

Serum CCL21 levels increased significantly after SCI. Moreover, serum CCL21 levels are significantly negatively correlated with cognitive function after SCI. After adjusting for confounding factors, the correlation between serum CCL21 and cognitive function after SCI still exists. These results suggest that serum CCL21 may be a potential biomarker for predicting cognitive impairment after SCI.

## Figures and Tables

**Table 1:** Demographics and clinical characteristics.

	NC (*n* = 49)	SCI (*n* = 84)	*p*
Age (years)	46.62 ± 4.98	47.31 ± 5.03	0.445
Gender (male/female)	35/14	56/28	0.569
BMI (kg/m^2^)	24.53 ± 1.66	24.92 ± 1.72	0.204
TG (mmol/L)	1.51 ± 0.19	1.54 ± 0.21	0.412
LDL-C (mmol/L)	2.43 ± 0.25	2.49 ± 0.28	0.218
FBG (mmol/L)	5.86 ± 0.57	5.90 ± 0.64	0.718
SBP (mmHg)	139.8 ± 12.3	141.1 ± 13.7	0.585
DBP (mmHg)	88.3 ± 9.4	90.5 ± 10.6	0.231
CCL21 (pg/ml)	41.3 ± 5.2	119.7 ± 13.5	<0.001
MoCA (points)	28.2 ± 1.2	24.6 ± 1.9	<0.001

NC: normal controls; SCI: spinal cord injury; BMI: body mass index; TG: triglycerides; LDL-C: low-density lipoprotein cholesterol; FBG: fasting plasma glucose; SBP: systolic blood pressure; DBP: diastolic blood pressure; CCL21: chemokine (C-C motif) ligand 21; MoCA: Montreal Cognitive Assessment.

**Table 2:** Relationship between serum CCL21 and MoCA.

Variable	Serum CCL21 levels (pg/ml)				*p*
	Q1	Q2	Q3	Q4	
MoCA (point)	25.7 ± 1.2	24.9 ± 1.5	24.0 ± 1.7	22.6 ± 2.0	0.023

CCL21: chemokine (C-C motif) ligand 21; MoCA: Montreal Cognitive Assessment.

**Table 3:** Multivariable analyses of risk factors with MoCA in SCI.

	Regression coefficient	*p*	95% CI
Age (years)	0.181	0.219	0.108-1.275
Male	0.346	0.393	0.298-1.201
BMI (kg/m^2^)	0.239	0.168	0.117-1.093
TG (mmol/L)	0.256	0.475	0.162-1.154
LDL-C (mmol/L)	0.266	0.311	0.143-1.138
FBG (mmol/L)	0.398	0.279	0.154-1.219
SBP (mmHg)	0.387	0.638	0.229-1.132
DBP (mmHg)	0.309	0.446	0.195-1.213
CCL21 (pg/ml)	0.328	0.027	0.202-0.807

MoCA: Montreal Cognitive Assessment; SCI: spinal cord injury; BMI: body mass index; TG: triglycerides; LDL-C: low-density lipoprotein cholesterol; FBG: fasting plasma glucose; SBP: systolic blood pressure; DBP: diastolic blood pressure; CCL21: chemokine (C-C motif) ligand 21.

## Data Availability

The data used to support the findings of this study are available from the corresponding author upon request.
